# P-579. Putting our Heads Together: Multidisciplinary Approach to Persons Living with HIV with Unsuppressed Viral Load

**DOI:** 10.1093/ofid/ofae631.777

**Published:** 2025-01-29

**Authors:** Ryan Rothman, Cassandra Oehler, George Bchech, Jasmine Moore, Chiu-bin hsiao

**Affiliations:** Allegheny General Hospital/Allegheny Health Network, Pittsburgh, Pennsylvania; Allegheny General Hospital, Positive Health Clinic, Center for Inclusion Health, AHN, Drexel University College of Medicine, Pittsburgh, Pennsylvania; Allegheny General Hospital/Allegheny Health Network, Pittsburgh, Pennsylvania; Allegheny Health Network, Pittsburgh, Pennsylvania; Allegheny General Hospital, Positive Health clinic, Center for Inclusion Health, AHN; Drexel University, College of Medicine, Pittsburgh, Pennsylvania

## Abstract

**Background:**

Advances in antiretroviral therapy such as the use of single tablet regimens, improvements in tolerability, medications with low resistance, and availability of long-acting injectable antiretroviral regimens have made HIV viral control an achievable goal for persons living with HIV (PWHIV), leading to a longer healthy life and significantly reduced risk of HIV transmission. However, there is a subset of patients (5-10% of PWHIV prescribed antiretroviral therapy) who have a persistently elevated viral load. We aimed to target these individuals for better HIV viral control.

**Figure 1:**
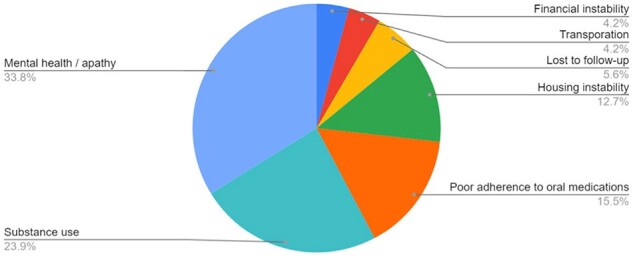
Barriers to HIV VL control The most common barriers encountered among the 38 patients discussed

**Methods:**

We identified patients from 2019 to 2024 in our Ryan White funded clinic with uncontrolled HIV, defined as a viral load (VL) greater than 200 copies/mL. Patients were placed in three categories: high risk (persistent VL > 200), vulnerable (inconsistent VL < 200), or stable (consistent VL < 200 for less than 2 years). We held weekly multidisciplinary meetings to discuss high risk and vulnerable patients and form an individualized plan of action. This plan was then discussed and enacted for each patient.

**Figure 2:**
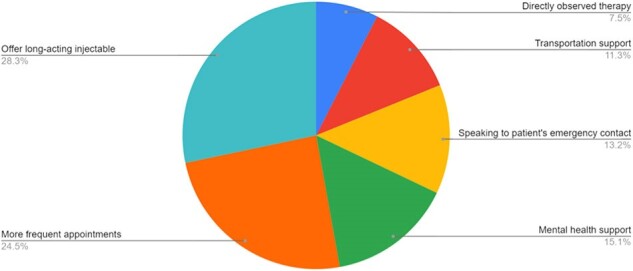
Interventions geared to overcome the barriers The most common interventions used among the 38 patients discussed to help overcome their unique barriers

**Results:**

73 patients were identified who fit into one of the three categories, including 39 high risk, 15 vulnerable, and 19 stable. Thus far, weekly meetings have been held for 38 of them. Figure 1 portrays the most common barriers to VL control, with many patients having multiple barriers. Each patient’s barriers were then addressed, with the most common interventions illustrated in Figure 2. Thus far, 11 patients have moved from the high risk group to the stable group, and 3 patients have moved from the vulnerable group to the stable group. VL trend from these 14 patients are shown in Figure 3.

**Figure 3:**
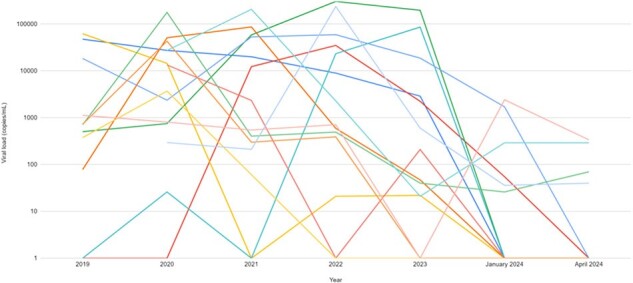
Viral load over time for 14 patients with improved HIV viral control Logarithmic graph of the viral loads over time for 14 patients who have moved from high risk or vulnerable to stable

**Conclusion:**

By identifying patients who were unable to bring their HIV VL under control and holding weekly multidisciplinary meetings to discuss specific barriers, we have been able to bring many high risk patients down to VL < 200. The most common barriers were related to mental health, substance use, and difficulty adhering to daily medications. The most common interventions were offering long-acting injectable regimens, increasing clinic visit frequency, and offering mental health support. We believe this multidisciplinary approach shows promise in decreasing the prevalence of unsuppressed HIV in clinical practice.

**Disclosures:**

**Chiu-bin hsiao, MD**, Viiv, Gilead: Grant/Research Support

